# Eco-Anxiety: An Evolutionary Line from Psychology to Psychopathology

**DOI:** 10.3390/medicina59122053

**Published:** 2023-11-21

**Authors:** Carmela Mento, Federica Damiani, Michele La Versa, Clemente Cedro, Maria Rosaria Anna Muscatello, Antonio Bruno, Rosa Angela Fabio, Maria Catena Silvestri

**Affiliations:** 1 Department of Biomedical and Dental Sciences and Morphofunctional Imaging, University of Messina, Via Consolare Valeria 1, Contesse, 98125 Messina, Italy; ccedro@unime.it (C.C.); mmuscatello@unime.it (M.R.A.M.); antonio.bruno@unime.it (A.B.); 2 Psychiatry Unit, Polyclinic Hospital University of Messina, Via Consolare Valeria 1, Contesse, 98125 Messina, Italy; federicadamiani9017@gmail.com (F.D.); michelelaversa2@gmail.com (M.L.V.); mariacate@libero.it (M.C.S.); 3 Department of Economics, University of Messina, Via dei Verdi 75, 98122 Messina, Italy; rosaangela.fabio@unime.it

**Keywords:** eco-anxiety, atmosphere, environmental factors

## Abstract

According to the scientific literature, climate change, due to human activities, can damage the environment, with psycho-physical consequences for humans. The scientific literature has highlighted how severe weather events can cause fear, stress, concern for the future, and eco-anxiety. In light of this information, this study aims to explore the concept of eco-anxiety. However, climate change is still perceived as a secondary problem. It would also be worth investigating the real importance that people attach to environmental issues compared to other circumstances, such as wars or pandemics.

## 1. Introduction

This work aims to explore the construct of eco-anxiety in the population and how much environmental concerns can contribute to the onset of this phenomenon. Previous studies have highlighted how environmental factors can influence the onset of possible psychopathologies, making people increasingly worried and anxious [1]. The idea that climate change is due to human activities, and that this change can harm us and future generations, could be closely related to the rise of a set of new, complex emotions that has gained more and more relevance in people’s everyday lives. Climate change is not just a matter of global warming: it is about all the new challenges that our ecosystem must face, threatening our mental health, such as the increasing risk of an extinction domino effect [2], the amplification of climate change threats like droughts, soil impoverishment with consequent starvation, and migration [3,4]. Describing the effects of climate change on mental health is one of the greatest challenges of the 21st century. However, it is not clear which possible subtypes of the syndromes defined in the literature are emerging, such as eco-anxiety, eco-guilt, and eco-pain, and how much suffering they can cause and to what extent they facilitate eco-sustainability behavior [5]. The term eco-anxiety is used to describe the anxiety felt in response to a stressful event related to the natural environment [6]. It could be considered a new topic due to its relevant impact on mental health and related physical effects. In fact, some studies have highlighted that there are some negative behaviors associated with eco-anxiety, such as irritability, sadness, depression, hopelessness, guilt, and anger [7]. As widely discussed on social media, climate change is producing disastrous effects on hydrological and terrestrial systems. However, climate change is also affecting our mental and physical health. According to previous studies, these sudden climate changes are generating different emotions in humans, including depression, anxiety, and anger [5]. 

Eco-anxiety is a type of stress and worry linked to the environmental crisis, characterized as concerns about climate change. Connected to this construct is eco-paralysis, i.e., the inability to significantly respond to climate and environmental challenges. An important phenomenon linked to eco-anxiety is eco-guilt, which occurs when people realize that they have violated personal or social standards of behavior [8]. Anxiety, unlike eco-anxiety, is referred to in the DSM-5 as a condition of tension that shows itself even for trivial reasons, with fear, apprehension, restless waiting, and often a series of physiological correlates such as tremors, sweating, palpitations, a sense of fatigue, and difficulty breathing normally [9]. The study by Agoston and colleagues (2022) interestingly shows six components of eco-anxiety: worries about the future and the next generations, empathy, conflict, psychological symptoms, loneliness, frustration, and feeling disturbed by uncontrolled and sudden climate changes [5]. In light of this information, this study aims to explore the psychological construct of eco-anxiety.

## 2. Materials and Methods

### 2.1. Research Strategy

The main purpose of this systematic review is to map empirical research concerning eco-anxiety and how it should be managed. 

This review was conducted according to the PRISMA methodological guidelines, which include searching for relevant studies, identifying the research question, and reporting the results [10]. This approach allowed us to incorporate several studies and highlight the importance of this topic. The criteria for inclusion and review procedures have been pre-specified in a protocol. The findings of this review are intended to contribute to higher awareness and better management of the new construct of eco-anxiety.

### 2.2. Identifying the Research Question

Various studies were identified in our search, including publications in peer-reviewed journals and qualitative information on eco-anxiety. Studies published in languages other than English and those irrelevant to the selected topic were excluded. Further exclusion criteria were articles, books, reviews, editorial comments, and case reports/series. 

### 2.3. Searching for Relevant Studies

The PubMed, Google Scholar, and EBSCO Discovery Service databases were searched from January 2013 to January 2023, using the following key terms: “eco-anxiety” OR “ecological anxiety” OR “eco-emotions”. The employed electronic search strategy is described in Table 1. 

The exclusion criteria were as follows: (a) comments, commentaries, opinions, letters to editors, interviews, specific editorials, conference abstracts or posters, book chapters, and books; (b) lacking details or quantitative information. Resources with these characteristics were not included in this review. In total, 1408 results were found. Subsequently, 360 studies were screened; according to the exclusion criteria, 343 studies were excluded as follows: 103 did not have full texts, 58 were not written in English, and 165 were not focused on the subject under study. The final number was 17 research studies that were thoroughly reviewed.

## 3. Results

The results of this review highlighted how climate change represents a possible threat to human mental health. Ongoing climate change exacerbates the concern for individuals’ wellbeing and the survival of humanity. The results of these studies show how considerable anxiety and concern about the possible expected negative impacts of climate change can be. Though few in number, increasing studies in the empirical literature have focused on the phenomenon of climate change anxiety (also known as eco-anxiety). However, it is still not clear how to manage it in psychological and clinical terms, as the studies examined so far describe the construct but do not clarify how to manage or prevent it. 

### The Landscape of Climate Emotions: Grief, Anger, and Guilt

As evident in the mentioned studies, a high level of emotional diversity is associated with climate change. It should be underlined that our review mainly focuses on the construct of eco-anxiety; in other words, it would be useful to dedicate further studies to other complex emotions linked to environmental issues. Below are some examples of the most prominent feelings, such as sadness, anger, frustration, and guilt, shown in the mentioned studies.

Numerous study participants and subjects experienced sadness and grief. These sad feelings were often connected to other emotions, such as feeling helpless and/or anxious about the future. Sadness was related to both the changes that had already occurred and the anticipated losses.

Ágoston et al. (2022) [5] recruited 17 participants via social media and used semi-structured interviews to explore people’s experiences regarding climate change. In the recruitment process, the authors did not apply rigid climatic criteria in the recruitment process but only considered the subjective experiences of the participants. This approach did not evaluate any direct climate risks; in particular, it did not include a quantitative evaluation of the phenomenon. The authors showed six eco-anxiety factors, eight types of eco-guilt, and two types of eco-grief [5]. Clissold et al. (2022) based their study on several datasets. Taking advantage of a combination of qualitative and quantitative tools, quantitative data were analyzed using SPSS, and qualitative data were analyzed using latent content analysis via NVivo. Latent content analysis is a technique used to code social data for their surface and underlying meanings so that hidden themes in the data can be revealed. The authors found that severe weather events, experiences of loss and change, a lack of future planning, and a sense of injustice elicited negative emotions, including fear, stress, anxiety, fatigue, sadness, pain, anger, frustration, helplessness, and worry [3]. Comtesse et al. (2021) introduced the concept of ecological pain and framed it in the context of mourning in the case of a mountaineer in Central Europe. The authors described how ecological pain can represent a risk to mental health [11]. Coppola and Pihkala (2023) used semi-structured interviews to explore participants’ emotions regarding the climate. The questions were mainly qualitative in nature and aimed at providing a personal perception of environmental issues. Furthermore, the authors did not rigorously ask every question to every participant, but considerable space was given to explore the emerging new themes regarding emotions towards the climate. The findings showed an association with climate emotions in young Americans: their emotions about climate change, such as anger, pain, and guilt, were very common [4]. Gunasiri et al. (2022) showed the positive impacts of the climate on feeling optimistic in quantitative and qualitative studies. Quantitative data analysis was performed with Excel and STATA 16.0 with descriptive analysis, and a qualitative data analysis was performed sequentially using thematic analysis techniques combined with inductive and deductive approaches [12]. Hajek et al. (2022) studied, in a sample of 3091 participants, the association between climate anxiety and loneliness, as well as perceived social isolation, and demonstrated that climate anxiety and loneliness were positively associated [13]. Heeren et al. (2022) assessed climate anxiety in a sample of 2080 participants using the Climate Anxiety Scale and showed that individuals with high values of climate anxiety had higher levels of eco-paralysis [14]. Heeren et al. (2023) recruited 874 participants and assessed climate anxiety using the Climate Change Anxiety Scale [15], demonstrating that people had considerable anxiety about climate change [15]. The study by Hogg and colleagues (2022) is interesting, as the authors developed and validated a brief four-dimensional measure of eco-anxiety. They highlighted how eco-anxiety is distinct from other psychological constructs, presenting multiple facets. Eco-anxiety, unlike anxiety as described in the DSM, includes more than just affective and behavioral symptoms. Furthermore, some dimensions of eco-anxiety are more stable over time than others. Four dimensions of ecological anxiety emerged in this study, which were distinct from stress, anxiety, and depression: affective symptoms, rumination, behavioral symptoms, and anxiety about one’s negative impact on the planet. This study supported eco-anxiety as a quantifiable psychological experience [16]. The study by Jones et al. (2023) suggested that those who focus more on negative information about climate change are less likely to engage in pro-environmental behavior. Given the small amount of research on eco-anxiety, it is not possible to generalize these findings [17]. Maran et al. (2021) highlighted that media exposure is one of the main factors that influences climate anxiety and vice versa [18]. Marsk et al. (2023) created a workshop led by young people that was aimed at finding new ways of understanding and responding to eco-emotions; these workshops offered a psychologically safe space to express and explore their knowledge, thoughts, and feelings about climate change via storytelling, visual images, and discussions about hoped-for futures. The authors showed that climate change topics, such as the multidimensional understanding of climate change, generated painful emotions [19]. Mouguiama-Daouda et al. (2022) studied anxiety, depression, and eco-anxiety in participants using the French version of the CAS, the Environmental Identity Scale, the Generalized Anxiety Disorder-7, and the Beck Depression Inventory-II and showed that eco-anxiety was positively associated with depression [20]. Nadarajah et al. (2022) highlighted the role of climate change-related anxiety in 369 young participants [21]. Qi et al. (2022) highlighted how eco-concern may be related to eating in 257 participants and examined whether it may contribute to later eating disorders; a significant difference was observed in concerns about climate change between women and men, with women showing greater levels of concern [22]. Wullenkord et al. (2021) highlighted how climate anxiety is positively related to need frustration and negatively with need satisfaction [23].

**Figure 1:**
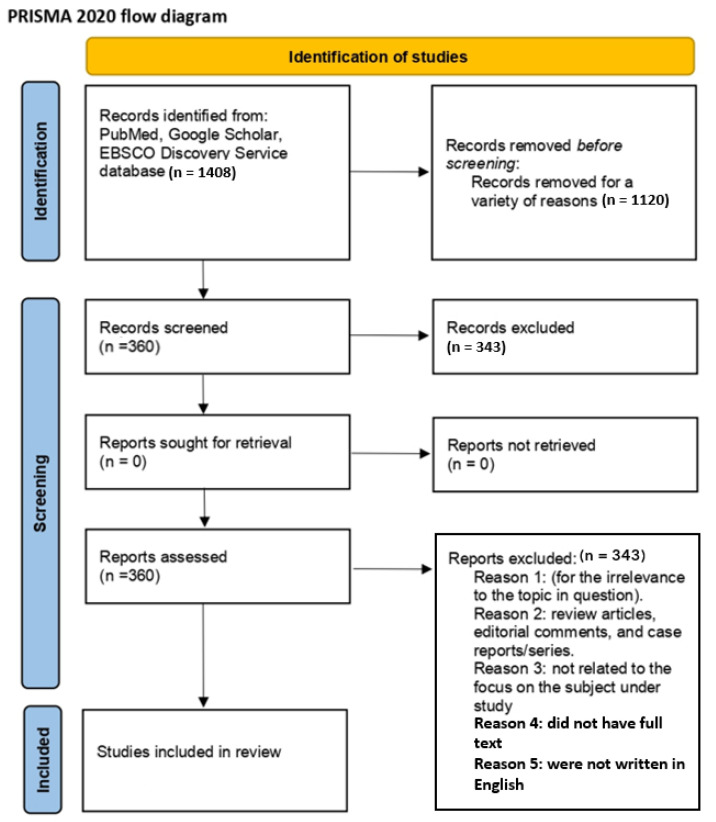
Flow diagram.

All data are reported in narrative format in the introductory section and discussion. Figure 1 summarizes the flowchart of the selected articles; Table 1 describes the keywords; Table 2 describes the aims, samples, and demographics of the population; Table 3 describes the findings of the included studies; and Table 4 describes the limitations and measurements of the included studies.

## 4. Discussion

This systematic review aimed at investigating the construct of eco-anxiety. Our research on the literature identified some empirical studies that discussed the impact of climate change on mental wellbeing among very different epidemiological clusters. For instance, some scholars have identified it as a feeling of anxiety related to anthropogenic climate change [16] or apprehension and stress about anticipated threats to ecosystems by climate change [24]. Other authors have interpreted it as a chronic fear of an unhealthy environment [25] or as a sensation felt when the ecological environment is collapsing. Within the field of climate psychology, the climate crisis poses profound threats in various ways, including threats to life and a sense of safety, life plans and expectations about the future, who we think we are as individuals and as a society, and our sense of worth, by challenging the morality of our destructive behaviors [26,27,28]. As a person learns about the reality of the climate crisis and the severity of its current, anticipated, and potential impacts, it is likely that such information will be perceived (consciously or unconsciously) as threatening, creating stress and triggering emotions such as anxiety, fear, worry, sadness, grief, despair, anger, shame, and guilt [29]. Maran and Begotti (2021) recently highlighted how concerns about climate change can elicit multiple emotional responses, including anger, sadness, desperation, fear, and guilt. Worry and anxiety are particularly common responses, often referred to as climate eco-anxiety. The authors analyzed how exposure to climate change is linked to climate anxiety and the sense of individual and collective self-efficacy [30]. Experiencing climate distress can be interpreted as a positive sign that the person is in touch with the reality of the climate crisis, which is necessary to generate appropriate responses [12,13]. Despite the diversity of the mentioned studies, in terms of the research methodology and sample, they all agreed in defining climate change as a serious threat to human mental health. Awareness of this threat may elicit eco-anxiety, which could be considered a rational and potentially adaptive response [17]. There is agreement over the fact that eco-anxiety is a psychopathological factor measurable with certain scales. Measurement can allow us to understand the impact of this problem on everyday life and give an idea of which coping strategies can be helpful depending on the severity of the case. Experiencing climate discomfort can be interpreted as a positive sign that the person is in touch with the reality of the climate crisis, which is necessary to generate appropriate responses [22,23,24]. Our review found that eco-emotions mostly affect younger age groups [31] and that eco-emotions are predominantly related to responses that do not induce practical anxiety, which leads to practical responses to problems. However, eco-emotions are also related to maladaptive coping, in which negative emotions and surrender responses prevail, generating feelings of emptiness and loneliness [3,4]. The key question is not so much about what particular distressing emotions are experienced, as people may experience a combination of these emotions simultaneously or fluctuate over time [16], but how emotions are regulated in the long term, whether suppressed, avoided, or otherwise dysregulated (maladaptive coping), or well tolerated and elaborated (adaptive coping). Avoidance or suppression coping strategies may be adaptive in the short term if a situation or context makes it unsafe for a person to interact with or express their feelings, but, in the long term, this is a form of maladaptive coping, with implications for health and personal aspects and climate action [23,24,31]. These results could be strengthened with evidence that the onset of some mental disorders can be associated with the experience of eco-anxiety.

## 5. Conclusions 

The topic of climate anxiety has been little studied empirically, so this study aimed at presenting the results of the mentioned studies, albeit with different methodologies, and offers some interesting insights for healthcare professionals (e.g., doctors, psychiatrists, and psychologists). However, research about ecological anxiety over the last decade has focused on a new type of psychological distress called eco-anxiety, and our findings lead us to recognize that further investigation is needed. In addition, it would be very useful to differentiate emotional consequences from ecological themes or patterns derived from catastrophes, wars, and pandemics. The results of this study can help professionals to gain a deeper understanding of the emotions related to climate change and how to adequately deal with them. 

## 6. Limitations

The limitations of this study arise from the limited literature so far, but this is probably due to the fact that eco-anxiety is a new construct. Another important limitation is the heterogeneous nature of these studies, in which there is a lack of methodological uniformity.

## Figures and Tables

**Table 1:** List of search terms used for identification of the studies for this systematic review.

Search Term
Eco-anxiety (all fields)
Ecological anxiety (all fields)
Eco-emotions (all fields)
1 OR 2 OR 3
English (language)
From January 2013 and January 2023 (publication date)

**Table 2:** Aims, samples, and demographics of populations included in this review.

References (Author, Place)	Aims	Sample	Demographics of the Study Population
[5]	This qualitative study aimed to evaluate the various forms of eco-anxiety.	17 participants were recruited via social media	-Residence: Hungary -Sex: 6 male and 11 female
[3]	The authors studied the variety of emotions that are expressed in Oceania as the Anthropocene unfolds.	72 participants	-Residence: Australia -Sex: female (71.4%) and male (28.6%)
[12]	This perspective article introduces the concept of ecological pain and considers it in the context of mourning.	One case description of a mountaineer in Central Europe	-Residence: Alpine region -Sex: male
[4]	This study examined the factors associated with climate emotions in young Americans.	14 participants	-Residence: U.S.A. -Sex: not specified
[11]	The aim of this study was to investigate the emotional aspects related to climate change in young Australians.	14 young people	-Residence: Australia -Sex: not specified
[13]	This study aimed to investigate aspects of climate anxiety in Germany.	3091 participants	-Residence: not specified -Sex: not specified
[14]	This study aimed to examine the effect of eco-anxiety.	2080 participants	-Residence: France, Belgium, Switzerland, Gabon, Rwanda, Algeria, Congo -Sex: not specified
[15]	The aim of this study was to examine the prevalence of climatic anxiety.	874 participants	-Residence: France, Belgium, Switzerland -Sex: women (413) and men (12)
[16]	This paper developed and validated a brief 4-dimensional measure of eco-anxiety.	334 participants	-Residence: Australia and New Zealand -Sex: not specified
[17]	The aim of the study was to explore the relationship between the experience of eco-anxiety and pro-environmental behaviors.	77 participants	-Residence: Western Australia -Sex: most participants identified as female (70.8%)
[18]	This article evaluated the effects of exposure to climate change information in the media in a sample of Italian university students aged between 18 and 26.	312 students	-Residence: Italy -Sex: women (74%) and men (25%)
[19]	This study aimed to find new ways of understanding and responding to eco-emotions.	The group was composed of three young collaborators (co-creators of the workshop), two facilitators (a clinical psychologist and a teacher), and four researchers (data analysis team).	-Residence: British -Sex: female (2) and male (2)
[20]	The objective was to identify the quantitative effects.	390 participants	-Residence: French -Sex: female (72.13%), male (26.89%), other (0.98%)
[21]	The aim of this study was to identify psychosocial factors of eco-anxiety.	369 participants	-Residence: French -Sex: not specified
[22]	The aim of this study was to investigate the possible relationship between eco-concern, the distress experienced in relation to climate change, and mental distress about one’s health status.	257 participants	-Residence: U.S.A. -Sex: female (44) and male (7)
[23]	This study investigated climate anxiety.	1134 participants	-Residence: Germany -Sex: female (51.14%) and male (43.91)

**Table 3:** Findings of studies included in this systematic review.

References (Author, Place)	Findings
[5]	This study showed six eco-anxiety factors, eight types of eco-guilt, and two types of eco-grief. The results showed data for the seventeen participants in ascending order based on the number of “psychoterratic” symptoms with the different types of coping mechanisms.
[3]	This study found that severe weather events, experiences of loss and change, a lack of future planning, and a sense of injustice elicited negative emotions, including fear, stress, anxiety, fatigue, sadness, pain, anger, frustration, helplessness, and worry. This sample had strong feelings of fear, stress, anxiety, and exhaustion as a result of acute and slow-onset weather events, and these impacts intensified and grew chronic due to repeated and prolonged disasters.
[12]	The authors described the ways in which ecological pain can occur, representing a risk to mental health, in a single case from an Alpine region.
[4]	The results highlighted very common emotions in regard to climate change, such as anger, pain, and guilt. Other emotions that participants linked to environmental issues included disappointment, fear, anxiety, despair, shame, uncertainty, and hope.
[11]	The findings of this study showed the positive impacts of the climate on feeling optimistic. The survey demonstrated that 43 (93%) participants thought “worry about the future” could be a mental health impact of climate change on young people.
[13]	This study demonstrated that climate anxiety and loneliness were positively associated. The association between climate anxiety and loneliness was r = 0.19 (*p* < 0.001).
[14]	These results showed that individuals with high values of climate anxiety had higher levels of eco-paralysis. There were no significant differences between participants from African and European countries.
[15]	This study demonstrated that people had considerable anxiety about climate change. Over 45% of the participants reported that their worries about climate change had detrimental consequences for their daily life functioning, notably because of their perception that their future was doomed.
[16]	Findings support eco-anxiety as a quantifiable psychological experience. Exploratory (*n* = 365) and confirmatory factor analysis (*n* = 370) supported a final 13-item scale that captured four dimensions of eco-anxiety, which were each distinct from stress, anxiety, and depression: affective symptoms, rumination, behavioral symptoms, and anxiety about one’s negative impact on the planet.
[17]	This study suggested that those who focus more on negative information about climate change are less likely to engage in pro-environmental behavior. Eco-anxiety was significantly positively correlated with depression.
[18]	These results highlighted that media exposure is one of the main factors that influence climate anxiety and vice versa. Most participants stated they paid either some or a lot of attention to information about climate change (89%), while only 11% stated that they paid little or no attention.
[19]	The following topics were identified: a multidimensional understanding of climate change, climate change generates painful emotions, and hope for the future. Sentiment classification was performed on the workshop transcripts to yield a positive percentage after the workshop.
[20]	Each factor of eco-anxiety was positively associated with depression.
[21]	This study highlighted role of anxiety related to climate change in young adults. The results show that information seeking predicts climate anxiety, which, in turn, predicts the emotional consequences of exposure to information about the negative consequences of climate change.
[22]	The results highlighted how eco-concern may be related to eating and whether it may contribute to later disordered eating. A significant difference was observed in climate change worry between female and male participants.
[23]	This study validated the core of the original CAS. On average, participants reported low climate anxiety (M = 1.81).

**Table 4:** Limitations and measurement types of studies included in this systematic review.

References (Author, Place)	Limitations	Type of Measurement
[5]	-Recruitment via social media. -Semi-structured interviews to explore people’s perceived experiences of climate change. -In the recruitment process, the authors did not apply rigid climatic criteria but only considered the subjective experiences of the participants. This approach did not evaluate some direct climate risks, lacking a quantitative evaluation of the phenomenon.	Semi-structured interviews to explore people’s experiences of climate change. The questions aimed to reveal associations, attitudes, emotions, behavioral intentions, and concrete behaviors related to climate change; the perceived effects of climate change; and sources of information on the topic.
[3]	-A possible limitation could be the socio-demographic divergence of the participants and the small sample size. In fact, among the 72 participants, the average age was 46 years (the youngest was 18, and the oldest was 78). Most participants indicated that they were “Caucasian” (30.6%), “Australian” (29.2%), “Anglo-Saxon” (6.9%), or had dual heritage.	-This study was based on several datasets. It used a mixture of qualitative and quantitative tools. Quantitative data were analyzed using SPSS, and qualitative data were analyzed using latent content analysis via NVivo. Latent content analysis is a technique used to code social data for its surface and underlying meanings so that themes in the data can emerge. -Sociodemographic data. -Livelihoods and values. -Story of disaster events and/or climatic changes.
[12]	-One case report.	-Case description.
[4]	-Semi-structured interviews to explore participants’ climate emotions. The questions were mainly qualitative in nature, aimed at providing a personal perception of environmental issues. Furthermore, the authors did not rigorously ask every question to every participant, but ample space was given to explore emerging themes in relation to climate emotions.	-Semi-structured interviews to explore climate emotions.
[11]	-Recruitment was conducted via social media. -A limited sample size to be able to generalize the results.	-Quantitative cross-sectional online survey about mental health impacts of climate change.
[13]	-The age of the interviewees ranged from 18 to 74 years.	-The De Jong Gierveld loneliness scale. -Bude and Lantermann tool. -The Climate Anxiety Scale.
[14]	-Online sample; due to this, a selectivity analysis could not be conducted.	-The Climate Anxiety Scale.
[15]	-Participants were recruited by a general community via online social media and listserv advertisements. -Age range between 18 and 81 years (uneven).	-The Climate Anxiety Scale.
[16]	Used mixed methods to explore eco-anxiety. Study was conducted on a sample originating from Australia and New Zealand, so the perception of climate change would certainly be different from the perception that an inhabitant of Africa, for example, might have.	-Tested a 7-item eco-anxiety scale.
[17]	-The sample was recruited in Australia and the speech language was only English.	-The Hogg Eco-Anxiety Scale. -Climate change beliefs. -Environmental self-efficacy. -Depression, Anxiety, and Stress Scale.
[18]	-A limited sample.	-The State-Trait Anxiety Inventory (STAI Y1). -The Perceived Climate Self-Efficacy Scale.
[19]	-The small sample size.	-A workshop was designed, led by young people, offering a psychologically safe space to express and explore their knowledge, thoughts, and feelings about climate change via storytelling, visual images, and discussion about hoped-for futures.
[20]	-Social media recruitment. -The participants were aged between 17 and 70.	-The Climate Anxiety Scale.
[21]	-Social media recruitment.	-Questionnaire consisting of climate anxiety; future consequences. -The Climate Change Anxiety Scale. -The 14-item Considerations of Future Consequences tool.
[22]	-Participants were recruited from across the United States. -Only inclusion criterion for the moment the study required individuals to be at least 18 years old; there were no exclusion criteria. -Online questionnaire survey.	-Eating-Related Eco-Concern. -The Climate Change Worry Scale. -The Eating Disorder Examination Questionnaire.
[23]	-Online survey. -When no validated German translations were available, the authors used back-translation to translate the measures.	-The Climate Anxiety Scale. -The PHQ-4. -The Climate Self-Protection Scale. -The System Justification Scale. -The Balanced Measure of Basic Psychological Needs Scale.

## Data Availability

The data are contained within the article.
